# Evidence That GH115 α-Glucuronidase Activity, Which Is Required to Degrade Plant Biomass, Is Dependent on Conformational Flexibility*

**DOI:** 10.1074/jbc.M113.525295

**Published:** 2013-11-08

**Authors:** Artur Rogowski, Arnaud Baslé, Cristiane S. Farinas, Alexandra Solovyova, Jennifer C. Mortimer, Paul Dupree, Harry J. Gilbert, David N. Bolam

**Affiliations:** From the ‡ Institute for Cell and Molecular Biosciences, The Medical School, Newcastle University, Newcastle upon Tyne NE2 4HH United Kingdom and; the § Department of Biochemistry, University of Cambridge, Tennis Court Road, Cambridge CB2 1QW, United Kingdom

**Keywords:** Bioenergy, Enzyme Structure, Glycoside Hydrolases, Plant Cell Wall, X-ray Crystallography

## Abstract

The microbial degradation of the plant cell wall is an important biological process that is highly relevant to environmentally significant industries such as the bioenergy and biorefining sectors. A major component of the wall is glucuronoxylan, a β1,4-linked xylose polysaccharide that is decorated with α-linked glucuronic and/or methylglucuronic acid (GlcA/MeGlcA). Recently three members of a glycoside hydrolase family, GH115, were shown to hydrolyze MeGlcA side chains from the internal regions of xylan, an activity that has not previously been described. Here we show that a dominant member of the human microbiota, *Bacteroides ovatus*, contains a GH115 enzyme, *Bo*Agu115A, which displays glucuronoxylan α-(4-*O*-methyl)-glucuronidase activity. The enzyme is significantly more active against substrates in which the xylose decorated with GlcA/MeGlcA is flanked by one or more xylose residues. The crystal structure of *Bo*Agu115A revealed a four-domain protein in which the active site, comprising a pocket that abuts a cleft-like structure, is housed in the second domain that adopts a TIM barrel-fold. The third domain, a five-helical bundle, and the C-terminal β-sandwich domain make inter-chain contacts leading to protein dimerization. Informed by the structure of the enzyme in complex with GlcA in its open ring form, in conjunction with mutagenesis studies, the potential substrate binding and catalytically significant amino acids were identified. Based on the catalytic importance of residues located on a highly flexible loop, the enzyme is required to undergo a substantial conformational change to form a productive Michaelis complex with glucuronoxylan.

## Teaser

**Background:** The structure of GH115 glucuronidases that remove glucuronic acid from xylan chains is unknown.

**Results:** *Bacteroides ovatus* GH115 glucuronidase is a dimeric enzyme that contains a flexible active site pocket.

**Conclusion:** The assembly of the catalytic apparatus of the glucuronidase requires substantial conformational changes.

**Significance:** Conformational changes are highly unusual in glycoside hydrolases.

## Introduction

The microbial degradation of plant biomass in soil is critical to the carbon cycle and is of increasing importance in environmentally significant industries, particularly the bioenergy and biorefining sectors, where the plant cell wall is the key substrate (1). Furthermore, dietary plant glycans that are not degraded by mammalian intestinal enzymes are the major nutrient available to the microbial communities of the fore-stomach or rumen in ruminants, and the large bowel in monogastric mammals (2). In humans the fermentation of dietary glycans by the microbes of the large bowel (defined as the microbiota) contributes up to 10% of daily calories (3). The microbiota also has a significant impact on the host through interactions with the immune system, which in turn can affect human health (see Ref. 4 for review). Plant cell walls present a highly complex substrate, and thus microorganisms that utilize these composite structures express an array of catabolic enzymes, primarily glycoside hydrolases, but also carbohydrate esterases and polysaccharide lyases, which degrade the structural polysaccharides into their component monosaccharides (see Refs. 5 and 6 for review). These enzymes are grouped into sequence-based families in the CAZy database (glycoside hydrolase and carbohydrate esterase families are defined as GH and CE, respectively) (7).

**FIGURE 1.**
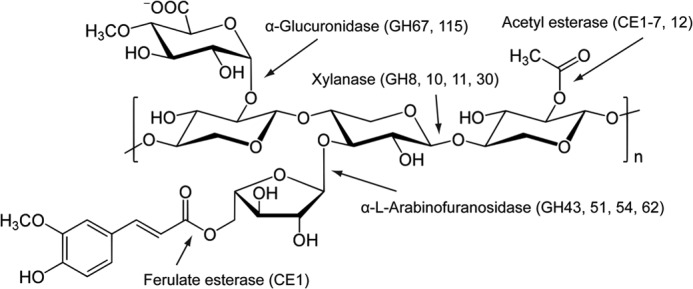
**Schematic of xylan.** The schematic shows a basic xylan structure, the enzyme classes that hydrolyze the various linkages, and their respective major CAZy families. Simple glucuronoxylans predominate in the walls of dicots, whereas monocots usually contain more complex glucuronoarabinoxylans.

Xylan, the major matrix polysaccharide of plant cell walls, consists of β1,4-linked d-xylose residues decorated at O2 and/or O3 with l-arabinofuranose and acetyl groups, and exclusively at O2 with α-d-glucuronic acid (GlcA) or 4-*O*-methyl-GlcA (MeGlcA) (Fig. 1) (8). These decorated regions make the polymer backbone recalcitrant to attack by most endo-xylanases and also block the action of xylosidases. To overcome this bottleneck, the side chains are removed by specific debranching enzymes, acetyl esterases, α-l-arabinofuranosidases, and α-glucuronidases (reviewed in Refs. 5 and 6). The majority of α-glucuronidases that hydrolyze the GlcA and MeGlcA decorations are located in GH67 (9, 10). These enzymes, however, do not attack glucuronoxylan, they only remove uronic acid from the non-reducing end of glucurono-xylooligosaccharides (9, 10). The structure of several GH67 enzymes show that O4 of the xylose decorated with GlcA is pointing at the surface of the enzymes, explaining why these α-glucuronidases can only target uronic acids attached to the non-reducing end of xylan chains (10, 11). Recently, two microbial eukaryotic enzymes from GH115 were shown to be α-glucuronidases that could cleave GlcA and MeGlcA from both the non-reducing end and internal xylose units of xylan and xylo-oligosaccharides (12, 13), displaying a single displacement acid base-assisted (inverting) mechanism, which results in inversion of the anomeric configuration of the cleaved uronic acid (14). The two enzymes had a higher specific activity against glucurono-xylooligosaccharides compared with glucuronoxylan, although no kinetic parameters for these enzymes were reported. As no crystal structure for a GH115 enzyme is available, the structural basis for the specificity of these α-glucuronidases is unknown.

To date, GH115 contains 237 members of which the majority (177) are bacterial, although only a single prokaryotic enzyme (from *Streptomyces pristinaespiralis*) has been shown to display α-glucuronidase activity (15). Bacteroidetes is a major phylum in the human gut microbiota. These organisms, exemplified by *Bacteroides ovatus*, are capable of degrading an extensive range of human and plant glycans (16). This glycan-degrading capacity is orchestrated by physically linked and co-regulated genes that are known as polysaccharide utilization loci (see Ref. 17 for review). The genome of *B. ovatus* contains seven open reading frames that are annotated as GH115s, two of which are in a polysaccharide utilization locus that is up-regulated during growth on complex xylans (16). Here we report the biochemical characterization and structure of one of these enzymes, BACOVA_03449, defined henceforth as *Bo*Agu115A. The enzyme was shown to be a glucuronoxylan-specific α-glucuronidase that displayed a strong preference for uronic acids that decorate internal xylose units. The enzyme displays the same core-fold as GH67 α-glucuronidases, however, the active site residues are not conserved in the two enzymes. Mutagenesis and structural data indicate that a significant conformational change is required to assemble the catalytic apparatus in *Bo*Agu115A.

## EXPERIMENTAL PROCEDURES

### 

#### 

##### Cloning, Expression, and Purification of GH115 Enzymes

The open reading frame encoding mature forms (and truncated derivatives of *Bo*Agu115A) of the seven *B. ovatus* GH115 proteins were amplified from genomic DNA (*B. ovatus* ATCC 8483) by PCR using primers that introduce NheI and XhoI restriction enzyme sites. The amplified DNA was cloned into the *Escherichia coli* expression vector pET28a (Novagen) such that the encoded recombinant proteins contain an N-terminal His_6_ tag. The recombinant proteins were produced in *E. coli* BL21 DE3 (Novagen) cells, harboring appropriate pET-based plasmids, and cultured in LB broth containing kanamycin (50 μg/ml) at 37 °C. Cells were grown to mid-exponential phase (*A*_600_ of 0.6), at which point isopropyl β-d-thiogalactopyranoside was added to a final concentration of 1 mm, and the cultures were incubated for a further 16 h at 16 °C. The cells were harvested by centrifugation, and His_6_-tagged recombinant protein was purified from cell-free extracts by immobilized metal affinity chromatography (IMAC) using standard methodology (18). For crystallographic studies *Bo*Agu115A was further purified by size exclusion chromatography using a Superdex 75 column. All proteins were purified to electrophoretic homogeneity as judged by SDS-PAGE.

##### Mutagenesis

Site-directed mutagenesis was conducted using the PCR-based QuikChange site-directed mutagenesis kit (Stratagene) according to the manufacturer's instructions, using the plasmid encoding *Bo*Agu115A as the template and appropriate primer pairs.

##### Nomenclature of Oligosaccharides

The oligosaccharides are defined as follows: for homopolymers of β1,4-linked xylose residues either the full name of the saccharide is used or the degree of polymerization is indicated as a subscript (*e.g.* the tetrasaccharide of xylose is denoted as xylotetraose or X_4_). Glucurono-xylooligosaccharides are identified by their sequence (unless otherwise stated) in which X is an undecorated xylose and U is a xylose containing an α1,2-linked [Me]GlcA (*e.g.* UXXX is xylotetraose in which the xylose at the non-reducing end contains an α1,2-linked [Me]GlcA).

##### Enzyme Assays

Substrates consisted of glucurono-xylans or glucurono-xylooligosaccharides. The glucurono-xylooligosaccharides UX and UXXX were purchased from Megazyme International (County Wicklow, Ireland), whereas UXX and XUXX were made as follows: 5 g of birch wood xylan (Sigma) was digested to completion with either *Cj*Xyn10A (19) or *Np*Xyn11A (20) xylanases in 50 mm sodium phosphate, 12 mm citrate buffer, pH 6.5, containing 1 mg/ml of BSA (PC buffer) to completion. The glucurono-xylooligosaccharides generated by *Cj*Xyn10A, UXX, or *Np*Xyn11A, XUXX, were purified from neutral xylooligosaccharides using Dowex chromatography (9). The structure of the products were confirmed by incubation with *Cj*GlcA67A, which generated xylotriose from the *Cj*Xyn10A product but no undecorated xylooligosaccharide from the *Np*Xyn11A product, whereas *Bo*Agu115A released xylotriose and xylotetraose from glucurono-xylooligosaccharides generated by the GH10 and GH11 xylanases, respectively. The deduced structure of the two glucurono-xylooligosaccharides is entirely consistent with the glucuronoxylan binding mode of the two xylanases (21, 22). Enzyme assays, in which polysaccharides or glucurono-xylooligosaccharides were the substrates, were carried out in PC buffer at 37 °C using enzyme purified to electrophoretic homogeneity by immobilized metal ion affinity chromatography. The concentration of enzyme varied from 10 nm for the wild type glucuronidase to 10 μm for the least active variants of *Bo*Agu115A. For kinetic assays, glucuronic acid was detected using the α-d-glucuronidase assay kit in which the uronic acid released is oxidized to glucarate with the concomitant reduction of NAD^+^ to NADH, which was monitored continuously at 340 nm and quantified using a molar extinction coefficient of 6220 m^−1^ cm^−1^. The molar concentration of the GlcA/MeGlcA in the glucuronoxylans was quantified by digesting 100 μg of the polysaccharides to completion with *Bo*Agu115A. To measure the activity of the *Bo*Agu115A mutants the xylotetraose reaction product released from XUXX was monitored by high performance anion-exchange chromatography (HPAEC) as described previously (18). The reaction was carried out in 20 mm sodium phosphate buffer, pH 7.0, at a substrate concentration (1 mm) that was well below the *K_M_*. Thus, the initial rate of hydrolysis of the glucurono-xylooligosaccharides gives a direct readout of *k*_cat_/*K_M_* (19). The glucuronidase-catalyzed reactions were also subjected to Polysaccharide Analysis using Carbohydrate gel Electrophoresis (PACE)^4^ as follows: a alcohol-insoluble residue was prepared from mature *Arabidopsis thaliana* wild type and *gux1gux2* stems as well as wild type willow, barley, sugar cane, and *Miscanthus* stems, as previously described (23). Alcohol-insoluble residue (500 μg) was pre-treated with 20 μl of 4 m NaOH for 1 h, neutralized with HCl, and ammonium acetate buffer, pH 6.0, added to a final concentration of 0.1 m and a final volume of 500 μl. The *Arabidopsis* alcohol-insoluble residue was digested to completion with xylanases (*Cj*Xyn10A and *Np*Xyn11A), a glucuronoxylanase (*Bo*GH30; Bacova_03432), and GH67 and GH115 α-glucuronidases (*Cj*GlcA67A and *Bo*Agu115A) as stated in the text and then dried in vacuum, whereas the other xylans were just digested with *Bo*Agu115A. Released mono- and oligosaccharides were labeled with 8-aminonaphthalene-1,3,6-trisulfonic acid (Invitrogen) and separated by gel electrophoresis as previously described (24). Xylooligosaccharides (Xyl_1–6_, Megazyme Int.) were derivatized alongside each set of samples and run as standards within each gel.

##### Crystallization, Data Collection, Structure Solution, and Refinement

SeMet-*Bo*Agu115A and native *Bo*Agu115A, at 10 mg/ml, were crystallized from 19% PEG3350, 0.2 m sodium citrate, pH 5.5. For both selenomethionine and native enzyme, crystals were harvested in a solution containing the mother liquor supplemented with 15% (v/v) PEG 400 or Paratone N oil as cryoprotectant and flash frozen in liquid nitrogen. Native crystals were soaked with 300 mm GlcA to obtain structures of *Bo*Agu115A in complex with the uronic acid product.

Diffraction data were collected at the Diamond Light Source, Didcot, United Kingdom, on beamlines I04–1 (ligand, λ = 0.9163 Å) and I02 (native and selenomethionine, λ = 0.9795 Å) at a temperature of 100 K. Data were processed and integrated with iMosflm (25) and scaled using SCALA (26). For all datasets, the space groups were determined to be P2_1_2_1_2_1_ with two molecules in the asymmetric unit (giving a Matthews coefficient of about 2.7 Å^3^ Da^−1^ and a solvent content of about 50%). Heavy atom sites were found and initial phases were calculated using the HKL2MAP interface for the ShelxC/D/E pipeline (27). All other computing used the CCP4 suite of programs (28). Phases were extended to an initial native dataset at 2.65 Å and a starting model was built using Buccaneer (29). The model underwent recursive cycles of model building in COOT (30) and refinement in REFMAC (31). The native model was used as the search model for molecular replacement in Molrep to solve the ligand datasets (32). Solvent molecules were added using COOT and checked manually. Five percent of the observations were randomly selected for the *R*_free_ set. The models were validated using Molprobity (33). The data statistics and refinement details are reported in Table 1.

**TABLE 1:** **Data statistics and refinement details**

	03449		
	Native	Selenomethionine	Ligand
**Data statistics***^a^*			
Beamline	IO2	IO2	IO4-1
Date	18/12/10	27/02/11	16/10/11
Wavelength (Å)	0.9795	0.9795	0.9173
Resolution (Å)	65.52-2.65	67.05-3.00	2.14
	(2.79-2.65)	(3.16-3.00)	(2.26-2.14)
Space group	P2_1_2_1_2_1_	P2_1_2_1_2_1_	P2_1_2_1_2_1_
Unit cell parameters			
*a* (Å)	75.39	76.13	72.04
*b* (Å)	131.68	132.75	130.29
*c* (Å)	199.40	201.14	190.17
α, β, γ (°)	90.0,90.0,90.0	90.0,90.0,90.0	90.0,90.0,90.0
Unit cell volume (Å^3^)	2,093,534	2,032,772	1,784,954
Solvent content (%)	56	54	48
No. of measured reflections	219,395	1,240,973	272,938
No. of independent reflections	56,839	41,710	93,249
Completeness (%)	97.5 (94.5)	100.0 (100.0)	94.4 (91.5)
Redundancy	3.9 (3.8)	29.8 (29.9)	2.9 (2.7)
*R*_merge_ (%)	12.6 (49.7)	19.8 (57.3)	9.6 (42.5)
〈I〉/〈σ(*I*)〉	8.5 (2.4)	20.2 (8)	9.3 (2.5)
Anomalous completeness (%)	NA	100.0 (100.0)	NA
Anomalous redundancy	NA	15.5 (15.4)	NA

**Refinement statistics*^a^***			
*R*_work_ (%)	20.34	NA*^b^*	17.40
*R*_free_*^c^* (%)	26.59	NA	21.91
No. of non-H atoms			
No. of protein, atoms	12888	NA	12695
No. of water molecules	23	NA	602
No. of other solvent atoms	2	NA	13
No. of ligand atoms	N/A	NA	13
Root mean square deviation from ideal values			
Bond length (Å)	0.09	NA	0.10
Angle distance (Å)	1.35	NA	1.62
Average *B* factor (Å^2^)			
Protein	31.5	NA	26.9
Ligand	NA	NA	36.9
Solvent water	22.5	NA	24.8
Solute ions (sodium)	26.5	NA	37.3
Ramachandran plot,*^d^* residues in allowed and most favored regions (%)	99.63	NA	99.81
Protein Data Bank codes	4C90		4C91

*^a^* Values in parentheses are for the highest resolution shell.

*^b^* N/A not applicable.

*^c^* 5% of the randomly selected reflections excluded from refinement.

*^d^* Calculated using MOLPROBITY.

##### Analytical Ultracentrifugation

Sedimentation velocity experiments were carried out in a Beckman Coulter (Palo Alto, CA) ProteomeLab XL-I analytical ultracentrifuge using interference optics. All analytical ultracentrifugation runs were carried out at the rotation speed of 48,000 rpm and experimental temperature of 20 °C; the velocity scans were taken 1 s apart, 600 scans in total. The sample volume was 400 μl. The rate protein sedimentation was used to calculate the *M*_r_ of the glucuronidase as described previously (34).

## RESULTS AND DISCUSSION

### 

#### 

##### BoAgu115A Is a Xylan-specific α-Glucuronidase

**FIGURE 2.**
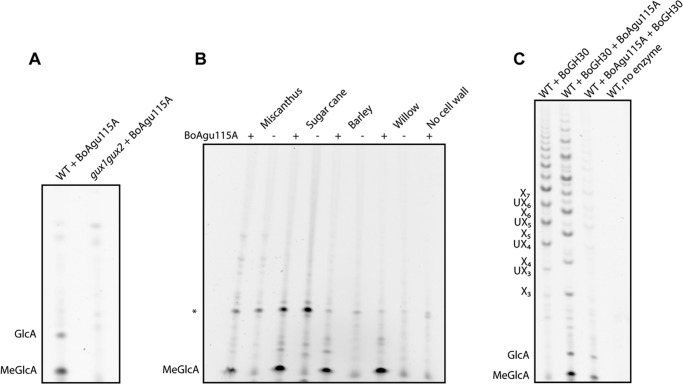
**The release of GlcA and MeGlcA from different xylans by *Bo*Agu115A.** In *panel A*, wild type (*WT*) *Arabidopsis* or the mutant *gux1gux2* (which does not decorate xylans with [Me]GlcA) were treated with *Bo*Agu115A. In *panel B*, the xylans extracted from the four plants were subjected to PACE in the absence (−) or presence (+) of *Bo*Agu115A. *, non-enzyme dependent product. In *panel C*, wild type *Arabidopsis* was treated with a GH30 glucuronoxylanase alone (WT + BoGH30), prior to treatment with *Bo*Agu115A (WT + BoGH30 + BoAgu115A), or subsequent to treatment with *Bo*Agu115A (WT + BoAgu115A + BoGH30). The glucurono-xylooligosaccharides displayed in *panel C* are as follows: *UX_3_*, XXUX; *UX_4_*, XXXUX; *UX_5_*, XXXXUX; *UX_6_*, XXXXXUX.

The genome of *B. ovatus* encodes seven proteins that are members of GH115. To explore their potential enzymatic activities, six of the seven *B. ovatus* GH115 proteins were expressed in *E. coli* in soluble form (BACOVA_00249 could not be expressed in *E. coli*), purified by IMAC to electrophoretic homogeneity, and their activity against glucuronoxylans was explored using PACE. The data showed that *Bo*Agu115A released glucuronic acid (GlcA) and 4-*O*-methyl-GlcA (MeGlcA) from wild type *Arabidopsis* glucuronoxylan, but not from an *Arabidopsis* mutant completely lacking MeGlcA and GlcA decorations on xylan (*gux1gux2*) (23) (Fig. 2*A*). The release of both GlcA and MeGlcA demonstrate that the enzyme can accommodate the 4-*O*-methyl substitution of GlcA, a trait shared with the other family of α-glucuronidases, GH67. The enzyme could also remove MeGlcA from glucuronoxylans present in bioenergy-relevant dicots such as willow, and from the more complex glucuronoarabinoxylans derived from monocots such as barley, sugar cane, and *Miscanthus* (Fig. 2*B*). Thus, *Bo*Agu115A is a α-glucuronidase that targets the uronic acids that decorate xylans. None of the other *B. ovatus* GH115 proteins exhibited measurable catalytic activity against a range of plant polysaccharides that contain uronic acids including glucuronoxylans, homogalacturonic acid, and rhamnogalacturonan I and II (data not shown). The lack of activity of one of these *B. ovatus* enzymes, BACOVA_03434, against glucuronoxylan is surprising as it is closely related to *Bo*Agu115A (54% identity), and is encoded by a gene within the same xylan-activated polysaccharide utilization locus. In contrast, BACOVA_00492, BACOVA_00982, BACOVA_02173, and BACOVA_02777 are more distantly related to *Bo*Agu115A and are not expressed in response to xylans, which may point to specificities that are not related to the hemicellulose.

##### Biochemical and Biophysical Properties of BoAgu115A

To explore the catalytic properties of *Bo*Agu115A in more detail the activity of the enzyme against glucurono-xylooligosaccharides and pre-treated glucuronoxylan was evaluated using PACE. When *Arabidopsis* glucuronoxylan was treated with the GH30 glucuronoxylan-specific xylanase, BACOVA_03432, a range of products were observed with the most prominent species having a degree of polymerization of 7 to 11. The GH30 enzyme was inactive against the hemicellulose that had been pre-treated with *Bo*Agu115A (Fig. 2*C*), indicating that glucuronidase can remove [Me]GlcA from all locations within the hemicellulose.

**FIGURE 3.**
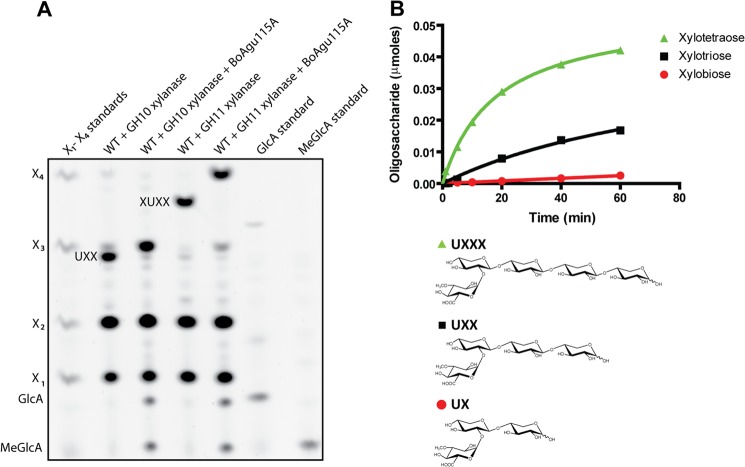
**Activity of *Bo*Agu115A against glucurono-xylooligosaccharides.** In *panel A*, wild type (*WT*) *Arabidopsis* glucuronoxylan was treated with either a GH10 or GH11 xylanase that produce terminal or internally substituted glucurono-xylooligosaccharides, respectively, as their end products. The enzymes were inactivated by heating and then subjected to PACE before and after treatment with *Bo*Agu115A. In *panel B*, a mixture of UX, UXX, and UXXX were incubated with *Bo*Agu115A and the product generated over time was monitored by the release of the appropriate xylooligosaccharide by high performance anion-exchange chromatography with pulsed amperometric detection (HPAEC-PAD).

Glucurono-xylooligosaccharides were generated by digesting *Arabidopsis* glucuronoxylan to completion (*i.e.* the product profile remains stable) with GH10 or GH11 xylanases, which generated UXX and XUXX as the limit products, respectively (35). The data showed that *Bo*Agu115A hydrolyzed UXX and XUXX (Fig. 3*A*), indicating that the enzyme cleaves GlcA from both non-reducing terminal and internal xylose residues, consistent with its activity against xylan, whereas the GH67 α-glucuronidase *Cj*GlcA67A released [Me]GlcA from UXX, but not from XUXX (data not shown), consistent with its capacity to remove the uronic acid only when it decorates the non-reducing terminal xylose (9, 11).

When *Bo*Agu115A was assayed against a mixture of terminally substituted aldouronic acids the activity of the enzyme followed the order UXXX>UXX≫UX (Fig. 3*B*), demonstrating that the enzyme contains at least two positive subsites downstream of the +1 subsite (+2R and +3R). Positive subsites bind to the xylan backbone with the +1 subsite housing the xylose linked to the cleaved GlcA, and subsites that bind successively to sugars toward the reducing end of the xylan chain are labeled +R2, +R3 etc., whereas the subsites that bind to xylose toward the non-reducing end are defined as +NR2, +NR3 etc. (nomenclature described in Ref. 36). It was also evident that *Bo*Agu115A was significantly more active against XUXX than UXX (Table 2), indicating that the glucuronidase contains at least one subsite upstream of the +1 subsite (+2NR). Against glucuronoxylans from birch and beech the *K_m_* of *Bo*Agu115A is ∼10-fold lower than XUXX, whereas the *k*_cat_ of the enzyme is ∼5-fold less against the polysaccharide compared with the oligosaccharide. Thus, although the α-glucuronidase has a higher catalytic efficiency against xylan than XUXX, its lower *k*_cat_ likely reflects tighter binding of the deglucuronylated xylan chains (*i.e.* reaction product) to the positive subsites, compared with the xylooligosaccharides released from the glucurono-xylooligosaccharides, resulting in slow product release and hence turnover rate. Thus, it is possible that the xylan binding site of *Bo*Agu115A may be able to bind more than four xylose residues.

**TABLE 2:** **Kinetics of wild type *Bo*Agu115A against glucuronoxylans and glucurono-xylooligosaccharides** Data are averages and S.D. from three independent experiments.

Substrate	*k*_cat_	*K_m_*	*k*_cat_/*K_m_*
	*min*^−*1*^	*mm*	*min*^−*1*^ *mm*^−*1*^
XUXX	3233 (±187)	4.5 (±0.54)	718
UXX	1106 (±71)	19.5 (±1.90)	57
Beech wood xylan	696 (±38)	0.4 (±0.06)	1740
Birch wood xylan	665 (±24)	0.3 (±0.04)	2217
Beech wood xylan /GH30*^a^*	837 (±77)	0.9 (±0.15)	930
Birch wood xylan/GH30*^a^*	862 (±64)	1.1 (±0.15)	784

*^a^* The polysaccharide was pre-digested to completion with a GH30 glucuronoxylan-specific xylanase.

**FIGURE 4.**
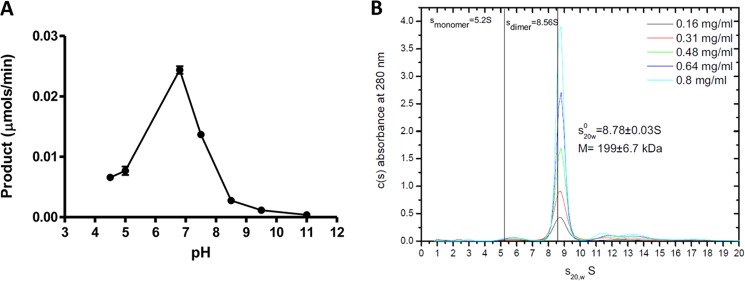
***Bo*Agu115A is a neutral acting dimeric α-glucuronidase.** In *panel A*, the enzyme was assayed against 1 mm XUXX in 50 mm sodium acetate, sodium HEPES, and sodium phosphate buffers at the different pH values. *Panel B* shows the analytical ultracentrifugation of *Bo*Agu115A.

The pH optimum of *Bo*Agu115A was ∼7.0 (Fig. 4*A*) and when subjected to analytical ultracentrifugation migrated with a sedimentation coefficient of 8.78 ± 0.03 S, which equates to a molecular mass of 199 ± 6.7 kDa (Fig. 4*B*). Given that the 824-amino acid recombinant form of *Bo*Agu115A has a molecular mass of 85 kDa, these data indicate that the glucuronidase is a dimer in solution, consistent with its crystal structure (see below).

##### Structure of BoAgu115A

**FIGURE 5.**
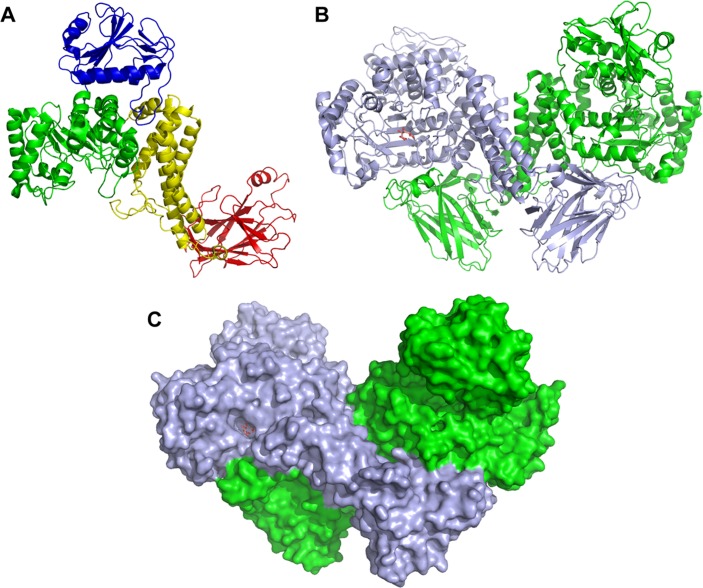
**Structure of *Bo*Agu115A.** *Panel A* is a schematic of a protomer of *Bo*Agu115A showing the four distinct domains of the enzyme: the N-terminal β-strand/α-helix domain (*blue*), the core TIM barrel catalytic domain (*green*), the five-helical bundle domain (*yellow*), and the C-terminal β-sandwich domain (*red*). *Panel B* depicts the enzyme in its dimeric butterfly-like form with protomers 1 and 2 colored *green* and *light blue*, respectively. *Panel C* shows a surface representation of the GH115 dimer. The relative orientation of the dimer and color of the protomers is as indicated in *B*. Both *panels B* and *C* show the GlcA bound form (sugar shown in stick format, carbons colored *salmon pink*).

The crystal structure of the native *Bo*Agu115A was solved by extending the phase information from an initial selenomethionine SAD at 3.0 Å to a native resolution of 2.65 Å, whereas the ligand structure of the enzyme was determined by molecular replacement at a resolution of 2.14 Å (Table 1). The final model of *Bo*Agu115A consists of one dimeric molecule in the asymmetric unit, with each protomer consisting of residues 33–856, with an average *B* factor of 31.5 (Table 1). The final crystallographic *R* value is 20.34, with an *R*_free_ of 26.59 for the native apo model, 17.40 and 21.91, respectively, for the ligand bound model. *Bo*Agu115A is an α/β globular protein with overall dimensions of about 100 × 70 × 50 Å per protomer. The enzyme consists of four distinct domains, which are connected by extended loops (Fig. 5*A*). The N-terminal domain, residues 33–196 (all residues are identified by their position in the full-length protein), comprises six β-strands that lay on top of two parallel α-helices. The second domain (amino acids 197–482) displays a (β/α)_8-fold_ (TIM barrel). This domain deviates slightly from a canonical TIM barrel; there is an additional helix between β-strands β-1 and β-2, and between β-3 and β-4, and the helices protruding from β-5 and β-6 are unusually short. The two α-helices of the N-terminal domain make extensive contacts with the α-helices extending from β-1 and β-2 of the TIM-barrel of the second domain. The third domain, extending from residues 488–641, comprises a five-helical bundle. The C-terminal domain, amino acids residues 673–844, displays a canonical β-sandwich-fold consisting of two β-sheets each containing five anti-parallel β-strands in the following order: β-sheet 1 (concave surface), β-1, β-8, β-3, and β-6; β-sheet 2 (convex surface), β-2, β-7, β-4, and β-5. In general the β-strands are connected by loops, however, the loop connecting β-1 and β-2 contains a pair of anti-parallel β-strands, whereas there is a short α-helix in the loop connecting β-5 with β-6. The two protomers in *Bo*Agu115A display a “butterfly” like structure in which the two subunits make several interactions through the helical bundle domains. In addition, the C-terminal β-sandwich domain of protomer-1 makes extensive interactions with the TIM-barrel domain of protomer-2 and vice versa (Fig. 5, *B* and *C*).

##### Identifying the Active Site of BoAgu115A

**FIGURE 6.**
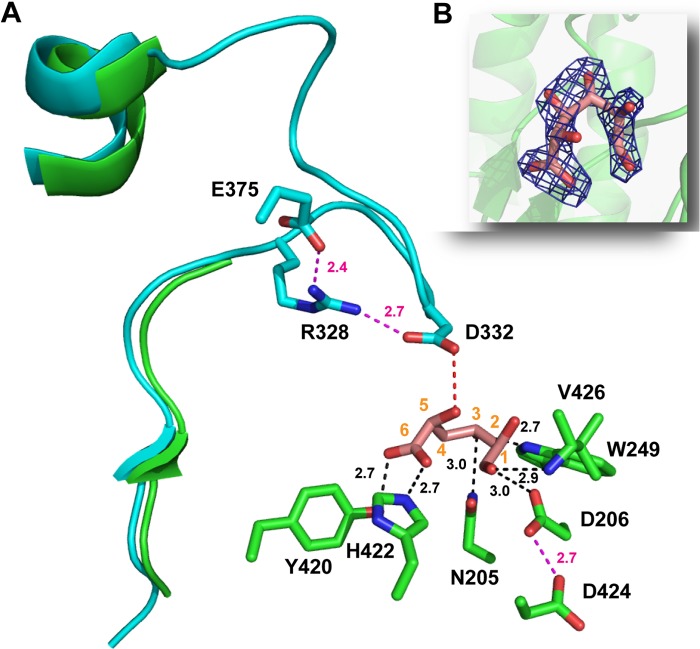
**The active site of *Bo*Agu115A.** *Panel A* shows the three-dimensional position of the amino acids (carbons colored *green* in the structure of the *Bo*Agu115A-GlcA complex and *cyan* in the apo structure) of *Bo*Agu115A that make polar interactions (indicated by *black dotted lines*) with bound GlcA (shown in stick format with carbons numbered and colored *salmon pink*) in its open ring conformation, or polar side contacts with other amino acids (indicated by *magenta dotted lines*). The distance in Å of these interactions are indicated. The *red dotted line* is a potential polar contact between Asp-332 and GlcA. The three-dimensional position of Arg-328, Asp-332, and Glu-375 are derived only from the apo structure of *Bo*Agu115A as the loop containing the aspartate and arginine and the side chain of the glutamate are disordered in the *Bo*Agu115-GlcA complex. The secondary structural elements shown in *green* and *cyan*, correspond to residues Glu-320 to Glu-345 of *Bo*Agu115A-GlcA and apo *Bo*Agu115A, respectively. The selected amino acids are shown in stick format with the carbons colored *green* (*Bo*Agu115A-GlcA) or *cyan* (apo *Bo*Agu115A) with all oxygens and nitrogens colored *red* and *blue*, respectively. *Panel B* shows the electron density map (2*F_o_* − *F_c_*) of GlcA at 1.3 σ. The electron density is shown in *dark blue*, protomer 1 is displays as a schematic in *green* and the atoms are colored as described in *panel A*.

**FIGURE 7.**
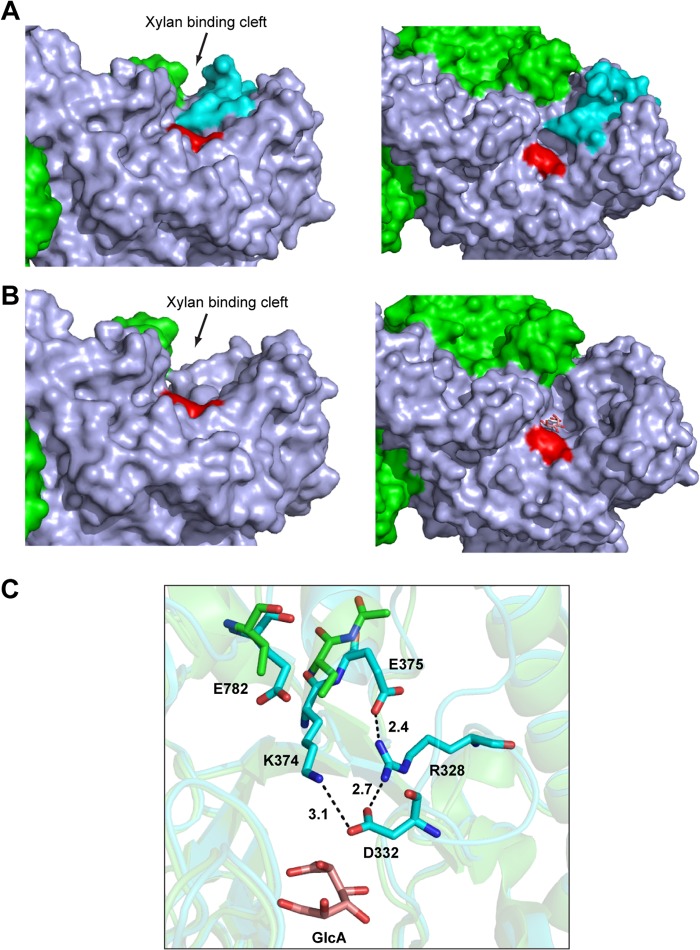
**The topology of the xylan binding cleft of *Bo*Agu115A.** *A* and *B* show a solvent-exposed surface representation of the substrate binding cleft of protomer 1 of the apo (*A*) and GlcA bound (*B*) forms of *Bo*Agu115A. In both panels the left-hand structures show a side on view down the putative xylan binding cleft (indicated with an *arrow*), and the right-hand structures provide a bird's eye view of the xylan binding cleft. In *A* and *B*, protomer 1 is colored *light blue*, protomer 2 is *green*, and the proximal wall (Trp-249) of the active site pocket is colored *red*. The flexible loop extending from Met-327 to Asn-346 is shown in *cyan* in *B* but not in *A* (the loop was too disordered to be modeled in the *Bo*Agu115A-GlcA complex). *C* shows the position of the amino acids that interact (Lys-374, Glu-375) with residues in the flexible loop (Asp-328, Asp-332) in stick format with the carbons (and schematic of the overall fold) colored *cyan* (apo *Bo*Agu115A) or *green* (*Bo*Agu115A-GlcA complex), the carbons of GlcA are colored *salmon pink*, and all oxygens and nitrogens colored *red* and *blue*, respectively. Note the side chains of Lys-374, Glu-375, and Glu-782 are too disordered in the apo structure to be modeled. The polar contacts between the amino acids are shown by *dotted lines* and the distance in Å of these interactions are indicated.

Inspection of the surface of the *Bo*Agu115A protomers revealed a cleft-like structure in the (β/α)_8_ domain that extends over the central β-barrel, on the opposite surface to the N-terminal domain. The center of the cleft, which may accommodate the xylan backbone, abuts onto a deep pocket that likely comprises the active site. This hypothesis was confirmed when *Bo*Agu115A was co-crystallized with GlcA, which revealed electron density for the uronic acid in the proposed active site pocket (Figs. 5, *B* and *C*, 6, and 7). The sugar was found to be in its ring open conformation, which is highly unusual for hexaose aldehyde sugars. It is possible that the specific interactions between the uronic acid and the enzyme encourage GlcA to adopt an open chain conformation after glycosidic bond cleavage. The direct interactions between GlcA and the enzyme are as follows (Fig. 6): O3 makes a polar contact with Nϵ-1 of Trp-249. O6A and O6B of the uronic acid carboxylate form polar contacts with the OH of Tyr-420 and Nϵ-2 of His-422, respectively, the endocyclic oxygen interacts with the N of Val-426, and O1 makes a hydrogen bond with Asp-206. O4 is pointing into a hydrophobic open channel, comprising the side chains of Phe-203, Met-248, and Trp-245, which likely make apolar contacts with the methyl group of MeGlcA. Unlike the vast majority of glycoside hydrolases, the active site of *Bo*Agu115A does not make typical parallel stacking interactions between the planar faces of aromatic residues and the sugar ring, although edge on edge apolar interactions between the sugar and two aromatic residues, Trp-249 and Tyr-420, and the aliphatic side chain of Val-426 were evident. The Oδ-2 of Asp-332 in the apo structure (the amino acid is not visible in the structure of the *Bo*Agu115A-GlcA complex, discussed in detail below), is in an appropriate position to make polar contacts with O2 of GlcA, whereas Oδ-1 of Asp-206 forms a polar contact with O1 and may therefore act as the catalytic acid (Fig. 6).

To explore further the significance of the interactions between GlcA and *Bo*Agu115A, and the function of other residues in the active site of the enzyme, targeted amino acids were substituted with alanine and the activity of the enzyme variants determined. The data, presented in Table 3 and Fig. 6, showed that mutating the residues that interact with the carboxylate of GlcA, His-422 and Tyr-420, resulted in a ∼10^5^- and ∼10^3^-fold reduction in activity, respectively, indicating that this component of the substrate is an important specificity determinant. O3 appears to interact with Trp-249 as the W249A mutation caused a ∼3000-fold reduction in catalytic activity. In addition to the residues that interact directly with the bound GlcA, several other amino acids in, or within the vicinity of, the active site were shown to make a significant contribution to the activity of the enzyme; the putative function of these residues is discussed below. Substituting amino acids near the putative xylan binding cleft/active site generally caused a modest reduction (5–100-fold) in the activity of the glucuronidase (see below), and mutating acid residues located in the other pocket/cleft-like structure on the enzyme (E162A, W169A, D192A, and D478A) did not affect activity (Table 3). These data are consistent with the view that the pocket located in the cleft-like structure in the TIM-barrel domain comprises the active site of *Bo*Agu115A.

**TABLE 3:** **Activity of mutants of *Bo*Agu115A**

*Bo*Agu115A derivative	Catalytic activity*^a^*
	*k_cat_/K_m_ min*^−*1*^ *m>m*^−*1*^
Wild type	780.0
E162A	∼780.0*^b^*
D192A	∼780.0*^b^*
W169A	∼780.0*^b^*
D478A	∼780.0*^b^*
E785A	198.0
Y788A	152.0
E782A	123.0
N205A	44.0
Y425A	10.8
K374A	8.0
Y792A	7.6
D396N	5.4
N398A	3.7
D206A	2.5
N462A	1.8
Y373A	1.1
Y420A	0.8
H275A	0.3
W249A	0.28
E375A	0.17
H422A	0.01
R328A	0.01
H275A/H422A	ND*^c^*
D332A	ND
ΔC-terminal (1–526)	ND
ΔC-terminal (1–639)	ND
ΔC-terminal (1–665)	ND

*^a^* Wild type and mutants of *Bo*Agu115A were assayed using 1 mm XUXX as the substrate in 20 mm sodium phosphate buffer, pH 7.0, containing 1 mg ml^−1^ of BSA. At this substrate concentration the initial rate provides a direct readout of the catalytic efficiency of the enzyme.

*^b^* ∼780; the activity of the mutants of residues from the other cleft/pocket-like structure on the enzyme was estimated from a single time point reaction.

*^c^* ND, no activity could be determined using an assay that can detect activity that is ∼10^5^-fold less than the wild type enzyme.

##### The Possible Assembly of the Catalytic Apparatus of BoAgu115A

GH115 enzymes are inverting glycoside hydrolase (14) and thus glycosidic bond cleavage is catalyzed by an acid base-assisted single displacement mechanism. In such a mechanism the catalytic Brønsted base, typically a carboxylate, activates a water molecule that attacks the anomeric carbon of the glycone sugar, whereas the catalytic acid, also a carboxylic acid residue, donates a proton to the glycosidic oxygen promoting leaving group departure (14). As stated above, Asp-206, based on its interaction with O1 of GlcA, may comprise the catalytic acid. Support for this view is provided by the apolar environment of the aspartate; the carboxylate amino acid is in close proximity with Trp-249 and Val-426, promoting an elevated p*K_a_*. Furthermore, Oδ-2 of Asp-206 is within hydrogen bonding distance with Oδ-1 of Asp-424, and thus may function as the p*K_a_* modulator of the putative catalytic acid (Fig. 6). It should be emphasized, however, that the assignment of Asp-206 as the catalytic residue must be viewed with some caution as the D206A substitution resulted in only a 300-fold reduction in activity, and, as the bound GlcA is in an open chain form, the position of O1 may not reflect the location of the atom when the uronic acid is in a closed pyranose configuration, adopted in the substrate.

Based on mutagenesis data the other carboxylate amino acids in the vicinity of the active site, Asp-332 and Glu-375, are candidate catalytic residues; the D332A and E375A mutations caused complete inactivation of the enzyme and a ∼5000-fold reduction in activity, respectively (Table 3). It is evident, however, that the distance between Asp-332 and Glu-375 (in the apo structure) and the O1 of GlcA (in the structure of *Bo*Agu115A in complex with GlcA); 5.4 Å and 11.8 Å, respectively, are greater than the normal ∼3.5 Å between the catalytic base and the anomeric carbon of the glycone sugar in inverting glycoside hydrolases (Fig. 6). It is possible that *Bo*Agu115A displays a “Grotthus”-style mechanism in which a remote amino acid activates the active site nucleophilic water via a string of solvent molecules (37), as proposed for some inverting glycoside hydrolases (37, 38). In such a mechanism the distance between the catalytic base and the sugar is not restricted to 3.5 Å. Both Asp-332 and Glu-375, however, are on highly mobile loops that have high *B*-factors. Indeed, in the *Bo*Agu115A-GlcA structure the loop that contains Asp-332 (Met-327 to Asn-346) was too disordered to be modeled, whereas Glu-375 could not be built past the β-carbon (Fig. 6). Thus, as discussed below, in the Michaelis catalytic complex Glu-375 and Asp-332 may adopt conformations, not observed in the apo and product structures, which reveal their true function in substrate binding and catalysis.

In enzymes and non-catalytic binding proteins that interact with glucuronic acid, arginines are often important specificity determinates, making bidentate hydrogen bonds/salt bridges with the carboxylate group (9, 39). It is possible that in *Bo*Agu115A Arg-328 contributes to substrate binding by also interacting with the carboxylate of the glucuronic acid substrate, a view supported by the observation that the R328A mutation caused ∼10^5^-fold decrease in activity (Table 3). Arg-328 (and Glu-375) in the apo structure, however, are too distant from the hub of the active site to play a direct role in enzyme function (Fig. 6). Thus, for the arginine to contribute to substrate binding the loop containing this amino acid (and Asp-332) would be required to undergo a conformational change. Another possibility is that the role of Arg-328 is to stabilize the conformation of Asp-332, which may function as the catalytic base, whereas the primary role of Glu-375 could be to orientate the guanidino group of the arginine toward the aspartate. To conclude, the above structural and mutagenesis data indicate that the assembly of the Michaelis complex in *Bo*Agu115A requires the repositioning of several flexible elements, most notably the loop carrying Arg-328 and Asp-332. Indeed, close inspection of the apo structure reveals that the conformation of this loop, which forms a component of the xylan binding cleft, in addition to part of the active site pocket, likely plays a central role in both glucuronoxylan binding and departure of the reaction products, both GlcA and the undecorated xylose polymer (Fig. 7, *A* and *B*). Furthermore, the loop also plays a role in stabilizing residues in the putative xylan binding cleft, notably Lys-374 and Glu-782, whose side chains are disordered in the GlcA bound structure where the loop is too flexible to be modeled (Fig. 7*C*). Although conformational changes are unusual in glycoside hydrolases, they are not without precedent, exemplified by the substantial movement of the catalytic TIM-barrel domain induced by substrate binding observed in GH112 glycoside phosphorylases (40).

Whether Asp-332, or possibly Glu-375, comprise the actual catalytic base is currently unclear, as is the role of Arg-328 in substrate binding, as a Michaelis complex could not be obtained. Although the catalytic apparatus of glycoside hydrolases generally comprise two carboxylate residues, exceptions are evident with histidine acting as a catalytic acid-base in a GH3 *N*-acetylglucosaminidase (41), and as a catalytic acid in an inverting GH117 anhydro-l-galactosidase (42). Given that alanine substitution of His-422 caused a ∼10^5^-fold reduction in activity, it is also possible that this residue is a component of the catalytic apparatus.

**FIGURE 8.**
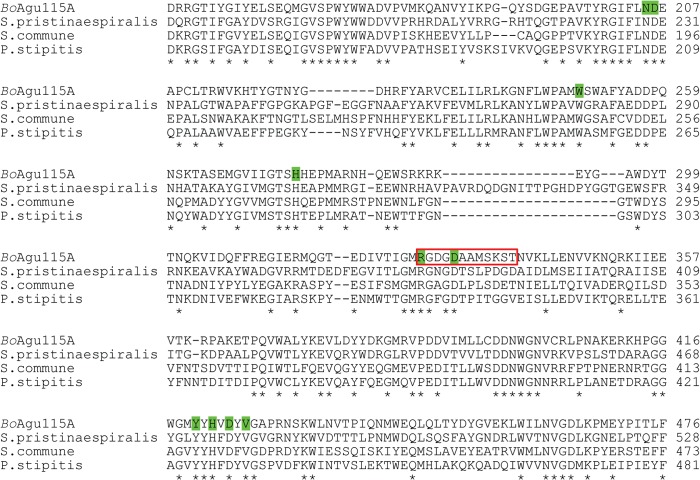
**Alignment of GH115 α-glucuronidases active against glucuronoxylan.** Only the amino acids that comprise the end of the N-terminal domain and the catalytic TIM-barrel domain of the *Bo*Agu115A are shown. Numbering in each protein is from the full-length native sequence. Identical residues in all sequences are indicated by an *asterisk*. Amino acids in *Bo*Agu115A highlighted in *green* are implicated in the catalytic activity of the enzyme. The flexible loop in *Bo*Agu115A that contains the key catalytic residues, Arg-328 and Asp-332, and is ordered only in the apo structure, is shown *boxed* in *red*. GenBank accession codes for the other characterized xylan-active GH115s shown are as follows: *Streptomyces pristinaespiralis*, EDY63299.2; *Schizophyllum commune*, ADV52250.1; and *Pichia stipitis*, ABN67901.2.

Sequence alignments showed that the residues that had the greatest influence on *Bo*Agu115A activity (mutants of amino acids that cause >10^4^-fold reduction in activity, those that interact with GlcA, and the proposed catalytic acid and p*K_a_* modulator, Asp-206 and Asp-424, respectively), are invariant in the other three GH115 enzymes that were shown to display α-glucuronidase activity (12, 13, 15) (Fig. 8). These data are entirely consistent with the proposed location of the *Bo*Agu115A active site, and the likely identity of the amino acids that contribute to catalysis.

##### The Potential Role of the C-terminal Domain of BoAgu115A

Inspection of the dimeric form of *Bo*Agu115A suggests that the C-terminal β-sandwich domain of protomer 2 contributes to one of the faces of the likely xylan binding cleft of protomer 1 and vice versa (Fig. 7). To explore the role of the C-terminal domain in the activity of the enzyme, alanine substitutions were made of Glu-782, Glu-785, Tyr-788, and Tyr-792, whose side chains line the proposed xylan binding cleft. The 100-fold reduction in catalytic activity mediated by the Y792A substitution (Table 3) indicated that Tyr-792 makes a significant contribution to xylan binding, whereas removal of the C-terminal domain (enzyme truncated at residue Ile-665) resulted in the complete abrogation of enzyme activity (Table 3), supporting a key role for this structural element in the topology of the GH115 catalytic apparatus.

##### Structural Comparison of GH115 and GH67 α-Glucuronidases

**FIGURE 9.**
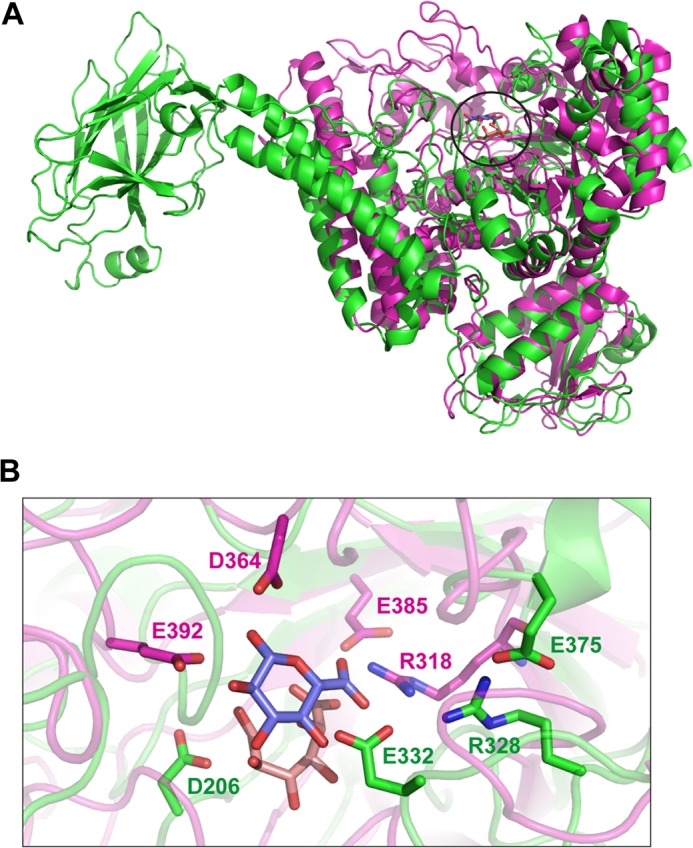
**Comparison of the active site of *Bo*Agu115A with a GH67 α-glucuronidase.** *Panel A* shows an overlay of a protomer of *Bo*Agu115A (*green*) with AguA, a GH67 α-glucuronidase (*magenta*; PDB code 1MQQ) from *G. stearothermophilus* (11). The position of the bound GlcA from *Bo*Agu115A and AguA are *circled. Panel B* shows an overlay of the active site of the two enzymes depicted in *panel A* in schematic format with the key catalytic and sugar binding residues displayed as *sticks*. The residues in which the carbons are colored *green* (apo form of *Bo*Agu115A; see Fig. 6 for the rationale for showing residues from the two structures) are from the GH115 enzyme and amino acids with carbons shown in *magenta* from the GH67 glucuronidase. The carbons of the GlcA in complex with *Bo*Agu115A and the GlcA bound to AguA are shown in *salmon pink* and *slate blue*, respectively, in both panels. All oxygens and nitrogens are shown in *red* and *dark blue*, respectively.

Structural comparison of *Bo*Agu115A with the PDB database (using DaliLite version 3) identified the *Geobacillus stearothermophilus* GH67 α-glucuronidase, AguA (11), as the closest, albeit weak, structural homolog with a *Z*-score of 16.1, root mean square deviation of 4.7 Å, and sequence identity of 11% over 470 aligned resides. Despite the weak structural homology and sequence similarity, the first three domains of the two proteins display the same fold and, intriguingly, the location of the active site of the GH115 and GH67 enzymes, endo- and exo-acting xylan-specific α-glucuronidase, respectively, is conserved, pointing to a distant evolutionary link between the two families (Fig. 9*A*). It is evident, however, that there is no conservation of the active site residues, although it should be noted that the catalytic acid in GH67 enzymes is positioned on a highly mobile loop that can only be stabilized in the presence of ligands (11), which has some resonance with the mobile loop containing the catalytically significant residues Arg-328 and Asp-332 in *Bo*Agu115A (Fig. 9*B*). In addition, the topology of the active site of the two enzymes is very different, comprising a deep pocket embedded in a rigid blind canyon (GH67 AguA) or a more accessible binding cleft (*Bo*Agu115A), consistent with the exo and endo specificities of the respective glucuronidases. In general the catalytic apparatus is conserved in enzymes that are structurally related and display the same substrate specificity, but exhibit distinct modes of action; the introduction of exo activity is mediated by the modification of the substrate binding cleft of endo-acting enzymes such that the cleft is converted into a blind canyon or a tunnel (reviewed by Refs. 5 and 6). It is intriguing, therefore, that despite significant structural conservation, indicating an evolutionary link between the two glucuronidase families, the region of the enzymes that are most likely to be highly conserved, the catalytic apparatus, is clearly different in GH67 and GH115. The evolutionary selection pressures that led to such diversity in the active site of structurally and functionally related enzymes families are currently unclear.

##### Conclusions

This report reveals the first structure of a GH115 enzyme, identifying a likely distant evolutionary link to GH67, the other major family of α-glucuronidases. The locations of the residues that contribute to activity indicate that the enzyme undergoes a substantial conformational change to assemble a functional catalytic apparatus. It is interesting to note that despite complete conservation of the catalytically important amino acids in seven GH115 proteins, only *Bo*Agu115A was shown to display activity (against glucuronoxylan). Although it is possible that the other six *B. ovatus* GH115 proteins are not catalytically active, a more likely explanation is that their target uronic acid-containing substrates are not glucuronoxylan, suggesting that GH115 is a poly-specific family.

## Footnotes

4 The abbreviation used is:

PACE: polysaccharide analysis using carbohydrate gel electrophoresis.
